# Association of Composite Dietary Antioxidant Index With the Risk of Nonalcoholic Fatty Liver Disease and All-Cause and Cause-Specific Mortality: Evidence From NHANES 2001–2018

**DOI:** 10.1155/ije/3255533

**Published:** 2025-04-18

**Authors:** Yingchao Ding, Miaomiao Fan, Chen Jiang, Naixin Dou, Yaqi Guo, Xiaolin Zhao, Xin Sun, Chunxiao Yu, Qingbo Guan

**Affiliations:** ^1^Department of Endocrinology, Shandong Provincial Hospital, Shandong University, Jinan, Shandong 250021, China; ^2^Department of Endocrinology, Shandong Provincial Hospital Affiliated to Shandong First Medical University, Jinan, Shandong 250021, China; ^3^Key Laboratory of Endocrine Glucose & Lipids Metabolism and Brain Aging, Ministry of Education, Jinan, Shandong 250021, China; ^4^Department of Endocrinology, Shouguang Peoples' Hospital, Shouguang, Shandong 262700, China; ^5^Department of Nuitrition, Shouguang Peoples' Hospital, Shouguang, Shandong 262700, China; ^6^Department of Health, Shandong University of Traditional Chinese Medicine, Jinan 250355, China

**Keywords:** composite dietary antioxidant index, mortality, NAFLD, NHANES

## Abstract

Evidence regarding the associations of composite dietary antioxidant index (CDAI) with the risk of prevalence and mortality of nonalcoholic fatty liver disease (NAFLD) is limited. We aimed to investigate these relationships using data from the National Health and Nutrition Examination Survey (NHANES). A total of 19,404 individuals with a mean age of 50.10 years were included. In multivariate-adjusted logistic regression analysis, compared to those in the lowest quartile of CDAI, individuals with the highest quartile were negatively associated with NAFLD prevalence (OR: 0.85, 95% CI: 0.75, 0.96), and the dose–response curve exhibited a linear relationship. Moreover, multivariate-adjusted Cox regression revealed that individuals with the highest quartile were negatively associated with cancer mortality (HR: 0.61, 95% CI: 0.37, 0.99) compared to those with the lowest quartile of CDAI in NAFLD patients. Moreover, the dose–response analysis demonstrated that CDAI had a nonlinear association with all-cause mortality in NAFLD patients. Multiple stratified and sensitivity analyses demonstrated that these associations are stable. CDAI was protective against the prevalence and cancer mortality of NAFLD. These results may provide new insights into adapting CDAI as a dietary strategy to prevent and improve the prognosis of NAFLD.

## 1. Introduction

Nonalcoholic fatty liver disease (NAFLD) is a clinical condition characterized by the accumulation of more than 5% lipids in hepatocytes without secondary causes of chronic liver injury, including viral hepatitis and excessive alcohol consumption [1]. NAFLD ranges from simple steatosis to more severe inflammatory and fibrotic forms of nonalcoholic steatohepatitis (NASH), with a small proportion progressing to cirrhosis or even hepatocellular carcinoma [2]. NAFLD is generally considered as the hepatic manifestation of metabolic syndrome and its prevalence is similar to obesity and Type 2 diabetes. At present, NAFLD is the most common chronic liver disease and the leading cause of liver transplantation in the world, with one-third of adults possibly suffering from NAFLD and is projected to increase in the future, imposing a heavy public health burden each year [3–5].

Dietary antioxidants, including antioxidants, vitamins, and minerals, have been documented to be protective against NAFLD/NASH, and even more advanced stages of fibrosis and HCC [6]. Recently, the composite dietary antioxidant index (CDAI) was developed to comprehensively assess dietary antioxidant intake [7], which is a combined score of several dietary antioxidants, including vitamins A, C, and E; selenium; zinc; and carotenoid, and reflects the total dietary antioxidant intake of individuals. Previous epidemiological evidence suggests that CDAI is associated with the onset and progression of multiple diseases, including cancers, hypertension, depression, all-cause mortality, and cardiovascular disease (CVD) mortality [8–12]. Recent studies have further highlighted the protective role of CDAI in various health outcomes. For instance, a higher CDAI has been shown to be a significant protective factor against chronic respiratory diseases (CRDs) and all-cause mortality in CRD patients, with the risk reduction being more pronounced in individuals with higher antioxidant intake. In addition, a higher intake of dietary antioxidants, as reflected by the CDAI, has been associated with a lower risk of fatty liver disease, particularly in women, suggesting a potential protective role in improving liver health and reducing the risk of metabolic dysfunction–associated liver diseases [13, 14]. However, little is still known regarding the association of CDAI with prevalence and mortality in NAFLD. A small-sample case-control study enrolling 295 patients with NAFLD showed that CDAI was negatively associated with NAFLD, suggesting a protective effect of CDAI against NAFLD [15]. Other observational studies with small samples have similarly shown that total dietary antioxidant capacity (TAC) is negatively associated with the prevalence of NAFLD [16–18]. Moreover, a cross-sectional study has reported that TAC is associated with lower hepatic ballooning (liver injury) in patients with NASH, yet there was no significant association between TAC and hepatic steatosis, lobular inflammation, and fibrosis [19].

To date, there is no epidemiological evidence unraveling the relationship between CDAI and mortality in NAFLD patients, and there is also a lack of large-sample studies verifying whether CDAI is associated with a reduced risk of NAFLD. In the current study, we utilized data from the National Health and Nutrition Examination Survey (NHANES), a large, nationally representative population-based study, to elucidate the relationship between CDAI and the prevalence and mortality of NAFLD.

## 2. Methods

### 2.1. Study Design

The NHANES database represents a national nutrition and health survey of the United States' population, which employs sophisticated, multistage, and stratified sampling methods. Details of the methods and protocols used in NHANES can be found on the CDC website (http://www.cdc.gov/nchs/nhanes.htm). The NHANES protocols received approval from the National Center for Health Statistics Research Ethics Review Board. All participants gave written informed consent to take part in the survey. The survey gathered data using household questionnaires, telephone interviews, and examinations carried out by medical professionals and trained staff.

This analysis included all participants from NHANES 2001–2018 of 91,351 and excluded the participants under the age of 18 (*n* = 37,595) and pregnant (*n* = 850) populations at first. Next, this study excluded people who lacked diagnostic indicators for CDAI (*n* = 6536) and fatty liver index (FLI) (*n* = 8231). In addition, this study excluded individuals with excessive alcohol consumption (*n* = 6809) and viral hepatitis (*n* = 623), and then excluded the individuals with missing covariate data (*n* = 11,303). Finally, 19,404 eligible participants for the analysis of CDAI and NAFLD prevalence and mortality were included, respectively. The flowchart of the study population is shown in Supporting Figure 1.

### 2.2. Dietary Recall Data

In NHANES, two 24 h dietary recall interviews were conducted: the first set of data was collected from participants at a mobile testing center, while the second set was gathered 3–10 days later through telephone consultation. To mitigate potential biases arising from individual dietary recall, this study used the average intake from both interviews. Individuals were asked for the details of food and beverages consumed in the previous 24 h. The mean intakes for each of the six antioxidants in the CDAI including vitamin A, vitamin C, vitamin E, zinc, selenium, and carotenoid were obtained. The CDAI was calculated according to the following formula based on the validated methodology established in previous studies, whereby each antioxidant intake was normalized by subtracting the mean intake and dividing by the standard deviation, and then summed all together to obtain the dietary antioxidant profile of an individual [20].

(1) $$\begin{array}{c} \text{CDAI}=\overset{6}{\underset{n=1}{\sum }}\frac{x-m\text{an}}{\text{SD}}. \end{array}$$

### 2.3. Definition of NAFLD

Since NHANES 2001–2018 does not have imaging modalities to diagnose NAFLD, this analysis utilized the FLI as a surrogate marker for NAFLD, FLI is a well-validated noninvasive score with a high diagnostic accuracy of 0.84. It was computed using serum triglycerides (TGs), body mass index (BMI), waist circumference (WC), and serum gamma–glutamyl transpeptidase (GGT) [21]. The FLI score of ≥ 60 was considered indicative of presumptive NAFLD.

(2) $$\begin{array}{c} \text{FLI}=\frac{e^{0.953*\log\text{⁣}e\text{⁣}\left(\text{TG}\right)+0.139*\text{BMI}+0.718*\log\text{⁣}e\text{⁣}\left(\text{GGT}\right)+0.053*\text{WC}-15.745}}{1+e^{0.953*\log\text{⁣}e\text{⁣}\left(\text{TG}\right)+0.139*\text{BMI}+0.718*\log\text{⁣}e\text{⁣}\left(\text{GGT}\right)+0.053*\text{WC}-15.745}}\times 100. \end{array}$$

### 2.4. Determination of Outcomes

In our study, we used NHANES 2001–2018 and prospectively linked it to the National Death Index mortality data with follow-up until December 31, 2019. The outcomes included all-cause, CVD, and cancer-related mortality. CVD mortality data were identified using ICD-10 codes I00-I09, I11, I13, I20-I51, or I60-I69, while cancer-related mortality was determined using ICD-10 codes C00-C97.

### 2.5. Statistical Analysis

Continuous variables (expressed as mean and standard error [SE]) and percentages of categorical variables were used to display the characteristics of the study population. The *t*-test for continuous variables or the chi-square test for categorical variables was used. Multivariable logistic regression analyses were used to evaluate the odds ratios (ORs) with 95% confidence intervals (CIs) for the association of CDAI with the prevalence of NAFLD in three models progressively adjusted for covariates. The initial model was unadjusted and did not account for any covariates. The multivariable-adjusted model accounted for age, gender (male or female), race/ethnicity (Mexican American, non-Hispanic Black, non-Hispanic White, other Hispanic, or other races), education (less than high school, high school, college, and above), marital status (married/living with partner or widowed/divorced/separated/never married), ratio of family income to poverty (PIR), as well as additional adjustments for smoking status (nonsmoker, former smoker, or current smoker), total sugar, total fat, protein, carbohydrates, dietary fiber, total energy intake, physical activity (metabolic equivalent [MET] < 600 min/week or MET ≥ 600 min/week), alcohol, total sugar, total fat, protein, carbohydrates, dietary fiber, cancer (no or yes), and comorbidities (no or yes). Participants with comorbidities are defined as the presence of at least two coexisting medical conditions, which include diabetes, stroke, CVD, and cancer.

Next, multivariable Cox regression analyses were used to evaluate the hazard ratios (HRs) with 95% CIs for the association of CDAI with mortality among patients with NAFLD. Similarly, a crude model without adjusting for any confounding variables was first constructed, followed by partially adjusted and fully adjusted models (adjusting for variables consistent with those in logistic regression, respectively). The restricted cubic spline (RCS) was performed to assess the potential nonlinear relationships between CDAI and the prevalence of NAFLD and mortality in patients with NAFLD. In addition, we conducted stratified and sensitivity analyses to confirm the stability of these associations. All analyses were conducted using R Version 4.2.2 (https://www.R-project.org). A *p* value < 0.05 (two-sided) was considered statistically significant.

## 3. Results

### 3.1. Baseline Characteristics

The characteristics of the study population are summarized in Table 1. The mean age was 50.10 ± 17.27 years, with 50.24% being female. Of these participants, 8687 were diagnosed with NAFLD based on the FLI. Participants with NAFLD tended to be older and had lower PIR and higher total energy intake compared to those without NAFLD. Importantly, both the CDAI and the six dietary antioxidants intake were lower in patients with NAFLD. Moreover, participants with NAFLD tended to be male, non-Hispanic Whites; former or current smokers; have lower education level; were characterized by a greater likelihood of being married/living with a partner; have higher intakes of total sugar, total fat, protein, and carbohydrates; and to have comorbidities such as diabetes, hypertension, and CVD.

### 3.2. Association Between CDAI and Prevalence of NAFLD

The relationships between six dietary antioxidants and the prevalence of NAFLD are shown in Supporting Table 1. After adjustment for confounding factors, dietary vitamin A, vitamin C, vitamin E, and carotenoid were both negatively associated with the prevalence of NAFLD. Dietary vitamin A (OR: 0.79, 95% CI: 0.72, 0.87), vitamin C (OR: 0.81, 95% CI: 0.73, 0.89), and vitamin E (OR: 0.84, 95% CI: 0.75, 0.94) at the Q4 group (compared to Q1) were associated with 21%, 19%, and 16% lower prevalence of NAFLD, respectively.

Table 2 shows the relationships between CDAI and the prevalence of NAFLD. After adjustment for confounding factors, we found that CDAI was negatively associated with the risk of NAFLD (OR: 0.97, 95% CI: 0.96, 0.99). The CDAI in the Q4 group (OR: 0.85, 95% CI: 0.75, 0.96) were significantly associated with a lower prevalence of NAFLD compared to the reference group (Q1).

### 3.3. Association Between CDAI and Risk of Mortality in NAFLD Patients

After a median follow-up time (interquartile range [IQR]) of 112.00 months (65.00–165.00) and 107.00 months (57.00–158.00), 1161 and 1053 deaths occurred in those without and with NAFLD, respectively. In terms of cause-specific mortality, there were 350 and 350 CVD–related deaths, as well as 275 and 278 cancer-related deaths, recorded in the non-NAFLD and NAFLD populations, respectively (Supporting Table 2). For the relationship between individual antioxidants and mortality in the NAFLD population (Supporting Table 3), we found that vitamin E (HR: 0.54, 95% CI: 0.38, 0.77) at the highest quartile was inversely associated with all-cause mortality compared to those with the lowest quartile. Similarly, vitamin E (HR: 0.61, 95% CI: 0.42, 0.89) and carotenoid (HR: 0.62, 95% CI: 0.44, 0.86) in the Q4 group (compared to Q1) were inversely associated with CVD mortality in multivariate-adjusted models. In the multivariable Cox regression model, a significant negative relationship between CDAI and cancer mortality was found (*p*_for⁣trend_ < 0.005). The CDAI at Q2 (HR: 0.66, 95% CI: 0.48, 0.92) and Q4 (HR: 0.61, 95% CI: 0.37, 0.99) were significantly associated with reduced cancer mortality compared to the lowest quartile. While CDAI was not associated with all-cause mortality and CVD mortality (Table 3).

### 3.4. Dose–Response Relationship Between CDAI and the Risk of NAFLD and Mortality

**Figure 1:**
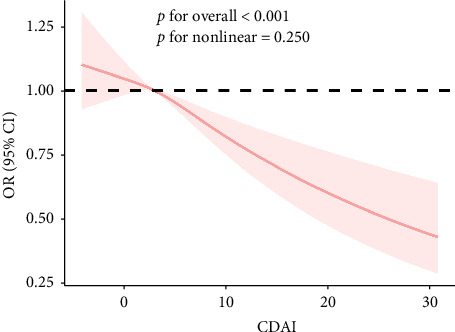
Dose–response association between CDAI and presence of NAFLD.

**Figure 2:**
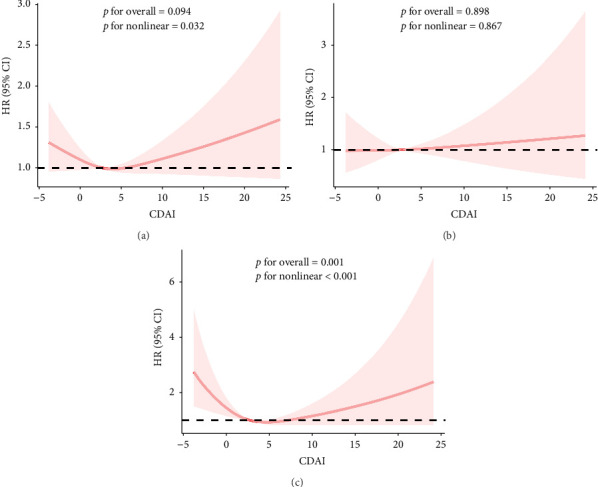
Dose–response relationship between CDAI and mortality from nonalcoholic fatty liver disease. (a) All-cause mortality. (b) CVD mortality. (c) Cancer mortality.

The RCS results showed a linear and inverse association between CDAI and prevalence of NAFLD (*p*_overall_ < 0.001, *p*_nonlinear_ = 0.250). Moreover, CDAI was nonlinearly associated with cancer mortality in the NAFLD patients (all-cause mortality: *p*_nonlinear_ = 0.032; cancer mortality: *p*_nonlinear_ < 0.001) but not with CVD mortality (*p*_overall_ = 0.867) (Figures 1 and 2).

### 3.5. Stratified and Sensitivity Analyses

Stratified analyses of the association between CDAI and NAFLD prevalence were performed based on age, sex, race, PIR, marital status, education level, smoking status, alcohol status, comorbidities, and physical activity. A negative association between CDAI and NAFLD was found across all of these subgroups (Supporting Table 4). We also conducted the sensitivity analysis excluding the participants whose time of death was within 2 years of the start of follow-up to examine the long-term reliability of the effect of CDAI on mortality in individuals with NAFLD (Supporting Table 5). In addition, by excluding specific subgroups (individuals with a history of alcohol consumption or cancer) or adjusting for key variables, the potential impact of these factors on the results was assessed (Supporting Tables 6 and 7). The findings were largely consistent with the overall results, further enhancing the credibility and robustness of the study conclusions.

## 4. Discussion

In the present study, the association of CDAI with the prevalence of NAFLD, as well as with all-cause and cause-specific mortality in NAFLD patients were assessed by using data from a population-based national survey. We found that CDAI was negatively associated with NAFLD prevalence and demonstrated a linear dose–response relationship. Furthermore, the results indicated that CDAI had a nonlinear relationship with both all-cause and cancer mortality but not with CVD mortality in NAFLD patients. Stratified analyses indicated the stability of the association between CDAI and the prevalence of NAFLD across different subgroups. Taken together, this study demonstrates the public health importance of CDAI in preventing NAFLD and for improving prognosis in NAFLD patients.

Our findings align with recent research highlighting the protective role of dietary antioxidants in various health outcomes. For example, a higher CDAI has been shown to significantly reduce the risk of CRDs and all-cause mortality in CRD patients, with the risk reduction being more pronounced in individuals with higher antioxidant intake. This underscores the broader health benefits of a diet rich in antioxidants, extending beyond liver health to respiratory and overall mortality outcomes. In addition, our results are consistent with studies showing that a higher intake of dietary antioxidants, as reflected by the CDAI, is associated with a lower risk of fatty liver disease, particularly in women. This suggests that dietary antioxidants may play a crucial role in improving liver health and reducing the risk of metabolic dysfunction–associated liver diseases [13, 14].

To our knowledge, the present study is the first to reveal an association of CDAI with the risk of NAFLD in a nationally representative sample and the first time that the association of CDAI with mortality has been explored in populations with and without NAFLD. There was only one case-control study with a small sample exploring the association of CDAI with the occurrence of NAFLD prior to our study [15]. This study from an Iranian population similarly indicated that CDAI was negatively associated with the risk of developing NAFLD, aligning with our findings. Of note, the CDAI used in this study consisted of dietary vitamin A, vitamin C, vitamin E, selenium, zinc, and manganese, slightly different from our included CDAI component. Dietary manganese intake data were not available in NHANES (instead, serum manganese information was provided), and therefore another common CDAI composition was used for exploration, which has been shown to be of significant clinical/public health importance in numerous epidemiological studies. Furthermore, our findings are supported by a recent study indicating that a higher intake of dietary antioxidants, as reflected by the CDAI, is associated with a lower risk of metabolic dysfunction–associated steatotic liver disease (MASLD) in United States' adults. However, no significant association was found between CDAI and advanced liver fibrosis, suggesting that while dietary antioxidants may reduce the risk of MASLD, their role in preventing fibrosis progression remains unclear. This highlights the need for further research to explore the mechanisms by which dietary antioxidants influence liver health and disease progression [22]. Notably, previous studies have shown that dietary antioxidants such as vitamin C, vitamin E, and selenium exhibit synergistic effects on health through their complementary roles in antioxidant defense, immune function, and cellular integrity [23, 24]. Furthermore, zinc and selenium are essential cofactors for antioxidant enzymes, such as superoxide dismutase (SOD) and glutathione peroxidase (GPx), which protect cells from oxidative damage. Taken together, these nutrients create a robust network that reduces oxidative stress, strengthens the immune system, and promotes overall health. Their interplay highlights the importance of a balanced diet rich in these micronutrients for optimal physiological function and disease prevention.

Real-world data suggested a systemic redox imbalance in NAFLD patients compared to healthy controls, and multiple serological markers reflecting oxidative stress were upregulated in NAFLD, indicating that oxidative stress may play a crucial role in the clinical pathogenesis of NAFLD [25]. However, there remains a scarcity of clinical studies that evaluate the effect of dietary antioxidant capacity on the onset and progression of NAFLD. Prior research has demonstrated that the oxidative balance score, which integrates pro-oxidants and antioxidants from diet and lifestyle, is negatively associated with the prevalence and incidence of NAFLD [26, 27]. A cross-sectional study enrolling 33 NASH patients indicated that a higher TAC may help reduce liver injury, although it did not improve hepatic steatosis and fibrosis [19].

The CDAI is a novel indirect measure of diet quality for assessing the integrated antioxidant potential of an individual's diet. We emphasize the importance of incorporating CDAI into public health campaigns and NAFLD–specific dietary recommendations to promote antioxidant-rich diets (e.g., the Mediterranean diet, which is high in fruits, vegetables, and whole grains). By doing so, healthcare providers can better guide patients in adopting dietary patterns that mitigate oxidative stress and inflammation, ultimately reducing NAFLD prevalence and improving liver health outcomes. The CDAI assesses the combined intake of individual dietary antioxidant micronutrients, which may also be associated with anti-inflammatory properties in addition to potential antioxidant properties [28, 29]. Previous observational studies have revealed that CDAI is linked to the development of several metabolic diseases, including obesity [30], diabetic nephropathy [31], and osteoporosis [32]. This study contributes new large-sample epidemiological evidence for the preventive role of CDAI in NAFLD.

A previous cohort study using NHANES showed that CDAI was correlated with a lower all-cause and CVD mortality in the general population [9]. Our study similarly found that CDAI was associated with lower all-cause mortality in NAFLD patients, but it may not be related to CVD mortality. One key difference in the previous study is that one of the components of CDAI used was manganese, rather than the carotenoid we used. Our study further showed that CDAI was nonlinearly associated with all-cause mortality in the NAFLD patients. Our findings provide new insights into the association of CDAI with mortality. Furthermore, CDAI has been reported to be associated with lower all-cause and/or factor-specific mortality in stroke [33], diabetes [31], and chronic kidney disease patients [34], suggesting a broad protective role for CDAI in diverse populations. Our study also indicated that CDAI exhibited a nonlinear association with cancer mortality in individuals with NAFLD. Few prior studies have assessed the association of CDAI with cancer mortality, and a previous cohort study showed that CDAI was not related to cancer mortality in people with diabetes [35]. Our findings suggest that CDAI may improve cancer mortality in patients with NAFLD.

An important finding was that stratified analyses revealed an interaction of smoking in the relationship between both CDAI and NAFLD risk and mortality in NAFLD patients. A previous study similarly demonstrated that smoking status influenced the relationship between CDAI and all-cause mortality in diabetic patients [35]. There are no studies exploring specific mechanisms, and we hypothesized that smoking-induced oxidative stress may impair blood levels of antioxidants [36], thereby altering the effects of CDAI. Moreover, the negative association of CDAI with NAFLD was presented only in the absence of diabetes, suggesting that diabetes has a compromising role on the NAFLD–preventive effect of CDAI and suggesting that CDAI be adjusted to diabetes status in clinical practice. Finally, high energy intake may impair the protective effect of CDAI against cancer mortality in NAFLD patients, suggesting that managing total energy intake could enhance the protection of CDAI against cancer-related mortality. Although there are no studies showing how CDAI exerts a protective effect against NAFLD, we can draw insights from improved oxidative stress and inflammation with dietary antioxidants. Overproduction of reactive oxygen species impairs cellular biological function, which can cause irreversible damage to cells through alterations in the immune microenvironment, inflammatory responses, and alterations to metabolism, including lipid peroxidation, ultimately leading to cell death [37]. Dietary intake of antioxidants including antioxidants, vitamins, and minerals has been associated with alleviation of oxidative stress in patients with NAFLD [38–41]. In this study, we found that individual antioxidants; vitamins A, C, and E; and carotenoids were both related to the lower prevalence of NAFLD and exhibited a dose–response relationship, whereas dietary selenium intake was associated with a higher prevalence of NAFLD. The present findings are consistent with a previous Mendelian randomization study suggesting that selenium may increase the risk of NAFLD, and that inflammation, insulin resistance, and oxidative stress induced by excessive selenium exposure may be responsible [42]. We determined that vitamin E was the component that most predominantly exerted a protective effect on mortality in subjects with and without NAFLD. A meta-analysis indicated that vitamin E may improve histological and biochemical parameters, steatosis, and fibrosis in patients with NAFLD [43]. Vitamin E is therefore one of the leading proven pharmacotherapies with therapeutic effects. Our study adds new evidence for the role of vitamin E on mortality in NAFLD patients, consistent with a previous study showing that vitamin E was negatively associated with all-cause mortality in NAFLD patients [44]. The CDAI, which integrates multiple antioxidants, offers a comprehensive approach to assessing dietary antioxidant intake and its association with NAFLD risk.

This study was based on the analysis of nationally representative NHANES with a large sample size and representatives. In addition, mortality data were obtained prospectively based on long-term follow-up, which reduced bias. In addition, this is the first to comprehensively explore the effect of CDAI on NAFLD prevalence and mortality in NAFLD patients, and the final conclusions were obtained through multiple unbiased statistical analysis methods, providing important public health implications. However, our study has shortcomings. In our study, we used FLI as surrogate markers for the diagnosis of NAFLD. However, these noninvasive indicators may lack accuracy compared to liver biopsy, particularly in distinguishing simple steatosis from NASH. Furthermore, although we adjusted for confounding variables where possible, residual confounders could still be present. In addition, cross-sectional design cannot establish the causal relationship between CDAI and NAFLD. Given the limited number of longitudinal studies currently available on CDAI and NAFLD, future investigations utilizing large-sample, prospective longitudinal cohort studies are warranted to further validate the findings of this study.

## 5. Conclusions

The present study suggests that CDAI was inversely associated with the prevalence of NAFLD and all-cause and cancer mortality in patients with NAFLD. These findings may offer new insights into the prevention and management of NAFLD through the modulation of CDAI.

## Figures and Tables

**Table 1:** Baseline analysis based on the presence of NAFLD, NHANES 2001–2018.

	Total (*n* = 19,404)	Non-NAFLD (*n* = 10,717)	NAFLD (*n* = 8687)	*p* value
Age, years	50.10 ± 17.27	48.46 ± 18.20	52.13 ± 15.82	< 0.001
Sex, *n* (%)				< 0.001
Male	9656 (49.76)	4816 (44.94)	4840 (55.72)	
Female	9748 (50.24)	5901 (55.06)	3847 (44.28)	
Race, *n* (%)				< 0.001
Mexican American	2602 (13.41)	1218 (11.37)	1384 (15.93)	
Non-Hispanic Black	3826 (19.72)	2016 (18.81)	1810 (20.84)	
Non-Hispanic White	9753 (50.26)	5489 (51.22)	4264 (49.08)	
Other Hispanic	1378 (7.10)	734 (6.85)	644 (7.41)	
Other race	1845 (9.51)	1260 (11.76)	585 (6.73)	
Marital status, *n* (%)				< 0.001
Married/living with partner	12,521 (64.53)	6758 (63.06)	5763 (66.34)	
Widowed/divorced/separated/never married	6883 (35.47)	3959 (36.94)	2924 (33.66)	
Education				< 0.001
< High school	1466 (7.56)	717 (6.69)	749 (8.62)	
High school	6314 (32.54)	3227 (30.11)	3087 (35.54)	
> High school	11,624 (59.91)	6773 (63.20)	4851 (55.84)	
Smoke				< 0.001
Never	11,426 (58.88)	6681 (62.34)	4745 (54.62)	
Former	5079 (26.18)	2423 (22.61)	2656 (30.57)	
Current	2899 (14.94)	1613 (15.05)	1286 (14.80)	
Alcohol, gm				
≤ 0	15,014 (77.38)	8065 (75.25)	6949 (79.99)	
> 0	4390 (22.62)	2652 (24.75)	1738 (20.01)	
Family income to poverty	2.82 ± 1.64	2.91 ± 1.65	2.71 ± 1.62	< 0.001
Body mass index kg/m^2^	28.76 ± 6.43	24.77 ± 3.36	33.69 ± 5.88	< 0.001
Waist circumference, cm	98.48 ± 15.78	88.17 ± 9.38	111.21 ± 12.42	< 0.001
Physical activity, MET min/week	3275.81 ± 5422.90	3251.53 ± 5356.70	3305.77 ± 5503.63	0.488
Composite dietary antioxidant index	3.57 ± 3.92	3.79 ± 4.13	3.30 ± 3.63	< 0.001
Vitamin A, mcg	649.77 ± 637.12	670.77 ± 695.55	623.85 ± 555.60	< 0.001
Vitamin C, mg	90.87 ± 82.28	94.64 ± 83.69	86.21 ± 80.27	< 0.001
Vitamin E, mg	8.09 ± 5.40	8.30 ± 5.71	7.83 ± 4.98	< 0.001
Zinc, mg	11.44 ± 6.82	11.34 ± 6.41	11.57 ± 7.29	0.018
Selenium, mcg	111.08 ± 53.21	109.94 ± 53.73	112.47 ± 52.53	0.001
Carotenoid, mcg	9950.62 ± 10,793.84	10,321.84 ± 11,486.50	9492.66 ± 9854.00	< 0.001
Energy intake, kcal/day	2049.53 ± 820.44	2038.39 ± 821.89	2063.27 ± 818.48	0.036
Protein, gm	81.32 ± 40.60	80.14 ± 40.14	82.78 ± 41.12	
Carbohydrates, gm	256.70 ± 123.98	256.21 ± 122.62	257.30 ± 125.64	0.542
Total sugar, gm	115.65 ± 77.73	115.30 ± 76.18	116.09 ± 79.61	0.483
Fiber, gm	17.27 ± 10.53	17.69 ± 10.79	16.75 ± 10.18	< 0.001
Total fat, gm	81.25 ± 45.25	79.30 ± 44.49	83.66 ± 46.06	< 0.001
Diabetes				< 0.001
No	16,366 (84.34)	9849 (91.90)	6517 (75.02)	
Yes	3038 (15.66)	8.68 (8.10)	2170 (24.98)	
Hypertension				< 0.001
No	11,513 (59.33)	7454 (69.55)	4059 (46.72)	
Yes	7891 (40.67)	3263 (30.45)	4628 (53.28)	
Cardiovascular disease				< 0.001
No	17,517 (90.28)	9930 (92,66)	7587 (87.34)	
Yes	1887 (9.72)	787 (7.34)	1100 (12.66)	
Cancer				0.002
No	17,471 (90.04)	9715 (90.65)	7756 (89.28)	
Yes	1933 (9.96)	1002 (9.35)	931 (10.72)	

*Note:* The continuous variables were presented as means ± standard error (SE). Categorical variables were expressed as the numbers and percentages. MET, metabolic equivalent.

Abbreviation: NAFLD, nonalcoholic fatty liver disease.

**Table 2:** Association between CDAI and the prevalence of NAFLD.

	Crude model	Model 1	Model 2
	OR (95% CI)	OR (95% CI)	OR (95% CI)
CDAI, continuous	0.98 (0.98, 0.99)	0.98 (0.97, 0.99)	0.97 (0.96, 0.99)

*CDAI, quartile*			
Q1	Reference (1.0)	Reference (1.0)	Reference (1.0)
Q2	1.02 (0.94, 1.10)	1.00 (0.92, 1.09)	0.99 (0.91, 1.08)
Q3	0.97 (0.90, 1.05)	0.94 (0.87, 1.03)	0.96 (0.87, 1.06)
Q4	0.89 (0.82, 0.96)	0.84 (0.77, 0.91)	0.85 (0.75, 0.96)
*p* for trend	< 0.0001	< 0.0001	0.0115

*Note:* Model 1 was adjusted for age, sex, race, marital status, PIR, and education, and Model 2 was further adjusted for smoking status, total sugar, total fat, protein, carbohydrates, dietary fiber, total energy intake, physical activity, alcohol, total sugar, total fat, protein, carbohydrates, dietary fiber, cancer, and comorbidities.

Abbreviations: CDAI, composite dietary antioxidant index; NAFLD, nonalcoholic fatty liver disease.

**Table 3:** Multivariable-adjusted Cox regression models revealing the relationship between CDAI and mortality in people with NAFLD.

	Crude model	Model 1	Model 2
	HR (95% CI)	HR (95% CI)	HR (95% CI)
*All-cause mortality*			
CDAI, continuous	0.94 (0.92, 0.96)	0.99 (0.97, 1.00)	1.00 (0.98, 1.03)

*CDAI, quartile*			
Q1	Reference (1.0)	Reference (1.0)	Reference (1.0)
Q2	0.90 (0.77, 1.04)	0.92 (0.79, 1.08)	1.01 (0.85, 1.19)
Q3	0.62 (0.52, 0.74)	0.78 (0.65, 0.92)	0.83 (0.69, 1.04)
Q4	0.57 (0.47, 0.68)	0.86 (0.72, 1.04)	0.99 (0.77, 1.27)
*p* for trend	< 0.0001	0.0196	0.4770

*CVD mortality*			
CDAI, continuous	0.94 (0.91, 0.97)	0.98 (0.95, 1.02)	1.01 (0.97, 1.05)

*CDAI, quartile*			
Q1	Reference (1.0)	Reference (1.0)	Reference (1.0)
Q2	0.90 (0.69, 1.17)	0.90 (0.69, 1.18)	1.02 (0.77, 1.36)
Q3	0.50 (0.36, 0.69)	0.80 (0.59, 1.08)	0.90 (0.64, 1.28)
Q4	0.50 (0.36, 0.69)	0.79 (0.56, 1.10)	0.94 (0.61, 1.46)
*p* for trend	< 0.0001	0.0894	0.6348

*Cancer mortality*			
CDAI, continuous	0.96 (0.92, 0.99)	0.99 (0.96, 1.03)	0.99 (0.94, 1.04)

*CDAI, quartile*			
Q1	Reference (1.0)	Reference (1.0)	Reference (1.0)
Q2	0.71 (0.52, 0.96)	0.71 (0.52, 0.97)	0.66 (0.48, 0.92)
Q3	0.67 (0.49, 0.92)	0.79 (0.58, 1.10)	0.82 (0.47, 1.00)
Q4	0.55 (0.39, 0.77)	0.77 (0.54, 1.09)	0.61 (0.37, 0.99)
*p* for trend	0.0005	0.1499	0.0433

*Note:* Model 1 was adjusted for age, sex, race, marital status, PIR, and education, and Model 2 was further adjusted for smoking status, body mass index, waist circumference, total sugar, total fat, protein, carbohydrates, dietary fiber, total energy intake, physical activity, alcohol, total sugar, total fat, protein, carbohydrates, dietary fiber, cancer, and comorbidities.

Abbreviations: CDAI, composite dietary antioxidant index; NAFLD, nonalcoholic fatty liver disease.

## Data Availability

This study analyzed publicly available datasets. The data can be found in the following website: https://www.cdc.gov/nchs/nhanes/.

## Supporting Information

Figure S1: The flowchart of the study population.

Table S1: Relationship between individual dietary antioxidants and the prevalence of NAFLD.

Table S2: Follow-up and mortality data.

Table S3: Association between individual antioxidants and mortality in the NAFLD population.

Table S4: Stratified analysis of the relationship between CDAI and NAFLD.

Table S5: Association of CDAI with mortality in the NAFLD population excluding those whose time of death was within 2 years of the start of follow-up.

Table S6: Association of CDAI with prevalence of NAFLD excluding those with a history of alcohol consumption.

Table S7: Association of CDAI with prevalence of NAFLD excluding those with cancer.
