# Supplementary material for: Association of Composite Dietary Antioxidant Index With the Risk of Nonalcoholic Fatty Liver Disease and All-Cause and Cause-Specific Mortality: Evidence From NHANES 2001–2018
Source: Int J Endocrinol. 2025 Apr 18;2025:3255533. doi: 10.1155/ije/3255533 (PMC12031604; doi:10.1155/ije/3255533)
Supplement: Supporting Information — Additional supporting information can be found online in the Supporting Information section. [file 3255533.f1.docx]

Figure S1. The flowchart of study population





Table S1. Relationship between individual dietary antioxidants and prevalence of NAFLD

|  | Crude model | Model 1 | Model 2 |
| --- | --- | --- | --- |
|  | OR (95% CI) | OR (95% CI) | OR (95% CI) |
| Vitamin A, continuous | 1.00 (1.00, 1.00) | 1.00 (1.00, 1.00) | 1.00 (1.00, 1.00) |
| Vitamin A, quartile |  |  |  |
| Q1 | Reference (1.0) | Reference (1.0) | Reference (1.0) |
| Q2 | 0.99 (0.91, 1.07) | 0.97 (0.90, 1.06) | 0.95 (0.87, 1.04) |
| Q3 | 0.87 (0.80, 0.94) | 0.85 (0.78, 0.93) | 0.85 (0.77, 0.93) |
| Q4 | 0.78(0.72, 0.85) | 0.76 (0.70, 0.83) | 0.79 (0.72, 0.87) |
| *P* for trend | <0.0001 | <0.0001 | <0.0001 |
| Vitamin C, continuous | 1.00 (1.00, 1.00) | 1.00 (1.00, 1.00) | 1.00 (1.00, 1.00) |
| Vitamin C, quartile |  |  |  |
| Q1 | Reference (1.0) | Reference (1.0) | Reference (1.0) |
| Q2 | 0.91 (0.84, 0.98) | 0.90 (0.83, 0.97) | 0.95 (0.87, 1.04) |
| Q3 | 0.82 (0.76, 0.89) | 0.81 (0.74, 0.87) | 0.88 (0.81, 0.97) |
| Q4 | 0.72 (0.66, 0.78) | 0.68 (0.63, 0.74) | 0.81 (0.73, 0.89) |
| *P* for trend | <0.0001 | <0.0001 | <0.0001 |
| Vitamin E, continuous | 0.98 (0.98, 0.99) | 0.98 (0.98, 0.99) | 0.98 (0.98, 0.99) |
| Vitamin E, quartile |  |  |  |
| Q1 | Reference (1.0) | Reference (1.0) | Reference (1.0) |
| Q2 | 0.98 (0.91, 1.07) | 0.99 (0.91, 1.07) | 0.98 (0.89, 1.07) |
| Q3 | 0.93 (0.86, 1.01) | 0.95 (0.87, 1.03) | 0.93 (0.85, 1.03) |
| Q4 | 0.84 (0.77, 0.91) | 0.85 (0.78, 0.93) | 0.84 (0.75, 0.94) |
| *P* for trend | <0.0001 | 0.0001 | 0.0027 |
| Zinc, continuous | 1.01 (1.00, 1.01) | 1.00 (1.00, 1.01) | 1.01 (1.00, 1.01) |
| Zinc, quartile |  |  |  |
| Q1 | Reference (1.0) | Reference (1.0) | Reference (1.0) |
| Q2 | 1.03 (0.95, 1.12) | 1.03 (0.95, 1.12) | 1.05 (0.96, 1.15) |
| Q3 | 1.04 (0.96, 1.13) | 1.01 (0.93, 1.10) | 1.05 (0.95, 1.16) |
| Q4 | 1.15 (1.06, 1.25) | 1.06 (0.97, 1.16) | 1.12 (0.99, 1.26) |
| *P* for trend | 0.0008 | 0.2579 | 0.0906 |
| Selenium, continuous | 1.00 (1.00, 1.00) | 1.00 (1.00, 1.00) | 1.00 (1.00, 1.00) |
| Selenium, quartile |  |  |  |
| Q1 | Reference (1.0) | Reference (1.0) | Reference (1.0) |
| Q2 | 1.08 (0.99, 1.17) | 1.08 (1,00, 1.18) | 1.10 (1.00, 1.20) |
| Q3 | 1.20 (1.11, 1.30) | 1.19 (1.10, 1.30) | 1.22 (1.10, 1.35) |
| Q4 | 1.21 (1.11, 1.31) | 1.18 (1.08, 1.29) | 1.21 (1.06, 1.38) |
| *P* for trend | <0.0001 | <0.0001 | 0.0005 |
| Carotenoid, continuous | 1.00 (1.00, 1.00) | 1.00 (1.00, 1.00) | 1.00 (1.00, 1.00) |
| Carotenoid, quartile |  |  |  |
| Q1 | Reference (1.0) | Reference (1.0) | Reference (1.0) |
| Q2 | 0.90 (0.83, 0.97) | 0.90 (0.83, 0.98) | 0.93 (0.85, 1.01) |
| Q3 | 0.82 (0.76, 0.89) | 0.83 (0.77, 0.90) | 0.89 (0.81, 0.97) |
| Q4 | 0.80 (0.74, 0.86) | 0.82 (0.75, 0.89) | 0.93 (0.85, 1.02) |
| *P* For Trend | <0.0001 | <0.0001 | 0.0825 |

The crude model did not adjust for any covariates, model 1 adjusted for some covariates including age, sex, race, marital status, PIR, and education, and model 2 was a fully adjusted model. NAFLD, non-alcoholic fatty liver disease.

Table S2. Follow-up and mortality data

|  | No-NAFLD | NAFLD |
| --- | --- | --- |
| Follow-up time, month(interquartile range, IQR) | 112.00 (65.00-165.00) | 107.0 (57.00-158.00) |
| All-cause death |  |  |
| No | 9546 (89.16) | 7628 (87.87) |
| Yes | 1161 (10.84) | 1053 (12.13) |
| Cause of death: CVD |  |  |
| No | 10367 (96.73) | 8337 (95.97) |
| Yes | 350 (3.27) | 350 (4.03) |
| Cause of death: cancer |  |  |
| No | 10442 (97.43) | 8409 (96.80) |
| Yes | 275 (2.57) | 278 (3.20) |

NAFLD, non-alcoholic fatty liver disease; CVD, cardiovascular disease.

Table S3. Association between individual antioxidants and mortality in the NAFLD population

|  | All-cause mortality | CVD mortality | Cancer mortality |
| --- | --- | --- | --- |
|  | HR (95% CI) | HR (95% CI) | HR (95% CI) |
| Vitamin A, continuous | 1.00 (1.00, 1.00) | 1.00 (1.00, 1.00) | 1.00 (1.00, 1.00) |
| Vitamin A, quartile |  |  |  |
| Q1 | Reference (1.0) | Reference (1.0) | Reference (1.0) |
| Q2 | 0.91 (0.65, 1.27) | 0.80 (0.57, 1.12) | 0.77 (0.55, 1.08) |
| Q3 | 1.04 (0.75, 1.44) | 0.78 (0.56, 1.09) | 0.76 (0.54, 1.08) |
| Q4 | 1.03 (0.74, 1.44) | 0.79 (0.56, 1.10) | 0.76 (0.52, 1.10) |
| *P* for trend | 0.6862 | 0.1768 | 0.1648 |
| Vitamin C, continuous | 1.00 (1.00, 1.00) | 1.00 (1.00, 1.00) | 1.00 (1.00, 1.00) |
| Vitamin C, quartile |  |  |  |
| Q1 | Reference (1.0) | Reference (1.0) | Reference (1.0) |
| Q2 | 0.93 (0.68, 1.26) | 0.80 (0.59, 1.10) | 0.82 (0.59, 1.12) |
| Q3 | 0.72 (0.51, 1.01) | 0.57 (0.40, 0.80) | 0.61 (0.43, 0.87) |
| Q4 | 0.82 (0.59, 1.14) | 0.75 (0.54, 1.04) | 0.84 (0.58, 1.21) |
| *P* for trend | 0.1067 | 0.0188 | 0.1413 |
| Vitamin E, continuous | 0.98 (0.96, 1.00) | 0.98 (0.95, 1.01) | 0.94 (0.90, 0.98) |
| Vitamin E, quartile |  |  |  |
| Q1 | Reference (1.0) | Reference (1.0) | Reference (1.0) |
| Q2 | 0.76 (0.56, 1.03) | 0.75 (0.55, 1.02) | 0.68 (0.49, 0.93) |
| Q3 | 0.69 (0.50, 0.95) | 0.71 (0.51, 0.98) | 0.58 (0.28, 0.73) |
| Q4 | 0.54 (0.38, 0.77) | 0.61 (0.42, 0.89) | 1.01 (1.00, 1.02) |
| *P* for trend | 0.0004 | 0.0062 | 0.0007 |
| Zinc, continuous | 1.00 (1.00, 1.01) | 1.01 (1.00, 1.01) | 1.01 (1.00, 1.02) |
| Zinc, quartile |  |  |  |
| Q1 | Reference (1.0) | Reference (1.0) | Reference (1.0) |
| Q2 | 0.97 (0.70, 1.36) | 0.94 (0.68, 1.32) | 0.88 (0.62, 1.25) |
| Q3 | 0.86 (0.61, 1.21) | 0.90 (0.63, 1.28) | 0.85 (0.57, 1.25) |
| Q4 | 0.82 (0.59, 1.14) | 0.98 (0.69, 1.40) | 1.00 (0.99, 1.00) |
| *P* for trend | 0.1770 | 0.8797 | 0.4808 |
| Selenium, continuous | 1.00 (1.00, 1.00) | 1.00 (1.00, 1.00) | 1.00 (1.00, 1.00) |
| Selenium, quartile |  |  |  |
| Q1 | Reference (1.0) | Reference (1.0) | Reference (1.0) |
| Q2 | 0.87 (0.63, 1.20) | 0.89 (0.64, 1.23) | 0.83 (0.59, 1.18) |
| Q3 | 0.73 (0.52, 1.02) | 0.84 (0.59, 1.18) | 0.77 (0.52, 1.15) |
| Q4 | 0.82 (0.60, 1.14) | 1.26 (0.89, 1.78) | 1.09 (0.66, 1.80) |
| *P* for trend | 0.1542 | 0.3239 | 0.9883 |
| Carotenoid, continuous | 1.00 (1.00, 1.00) | 1.00 (1.00, 1.00) | 1.00 (1.00, 1.00) |
| Carotenoid, quartile |  |  |  |
| Q1 | Reference (1.0) | Reference (1.0) | Reference (1.0) |
| Q2 | 0.63 (0.45, 0.87) | 0.60 (0.43, 0.83) | 0.59 (0.43, 0.82) |
| Q3 | 0.73 (0.53, 0.99) | 0.73 (0.53, 1.00) | 0.76 (0.55, 1.05) |
| Q4 | 0.60 (0.43, 0.83) | 0.62 (0.44, 0.86) | 0.62 (0.44, 0.89) |
| *P* for trend | 0.0041 | 0.0108 | 0.0248 |

NAFLD, non-alcoholic fatty liver disease; CVD, cardiovascular disease.

Table S4. Stratified analysis of the relationship between CDAI and NAFLD

|  | Total  (n = 19,433) | OR(95%CI) |
| --- | --- | --- |
| Age, year |  |  |
| <60 | 12834 | 0.97（0.96，0.98） |
| ≥60 | 6570 | 0.96（0.95，0.98） |
| Sex, n (%) |  |  |
| Male | 9656 | 0.97（0.96，0.98） |
| Female | 9748 | 0.97（0.95，0.98） |
| Race, n (%) |  |  |
| White | 2602 | 0.99（0.97，1.01） |
| Non-White | 16802 | 0.96（0.96，0.97） |
| Marital Status, n (%) |  |  |
| Married/living with partner | 12521 | 0.97（0.96，0.98） |
| Widowed/divorced/separated/never married | 6883 | 0.97（0.95，0.98） |
| Education |  |  |
| Less than high school | 1466 | 1.00（0.97，1.03） |
| More than high school | 17938 | 0.97（0.96，0.97） |
| PIR |  |  |
| ≤1 | 3102 | 0.96（0.94，0.98） |
| >1,≤3 | 7615 | 0.97（0.96，0.99） |
| >3 | 8687 | 0.97（0.96，0.98） |
| Smoke |  |  |
| Never | 11426 | 0.97（0.96，0.98） |
| Past or current | 7978 | 0.97（0.96，0.99） |
| Alcohol |  |  |
| ≤0 | 15014 | 0.97（0.96，0.98） |
| >0 | 4390 | 0.95（0.94，0.97） |
| Energy |  |  |
| High | 9702 | 0.94 (0.93, 0.96) |
| Low | 9702 | 0.95 (0.94, 0.96) |
| Comorbidities |  |  |
| No | 10251 | 0.97 (0.96, 0.98) |
| Yes | 9153 | 0.99 (0.98, 1.00) |

NAFLD, non-alcoholic fatty liver disease; CDAI, Composite dietary antioxidant index.

Table S5. Association of CDAI with mortality in the NAFLD population excluding those whose time of death was within two years of the start of follow-up

|  | Crude model | Model 1 | Model 2 |
| --- | --- | --- | --- |
|  | HR (95% CI) | HR (95% CI) | HR (95% CI) |
| All-cause mortality |  |  |  |
| CDAI, continuous | 0.93 (0.91, 0.96) | 0.96 (0.93, 0.98) | 0.99 (0.96, 1.03) |
| CDAI, quartile |  |  |  |
| Q1 | Reference (1.0) | Reference (1.0) | Reference (1.0) |
| Q2 | 0.80 (0.62, 1.02) | 0.93 (0.72, 1.20) | 0.98 (0.74, 1.29) |
| Q3 | 0.59 (0.46, 0.77) | 0.68 (0.52, 0.89) | 0.79 (0.58, 1.09) |
| Q4 | 0.51 (0.39, 0.67) | 0.66 (0.50, 0.87) | 0.91 (0.62, 1.32) |
| *P* for trend | <0.0001 | 0.0004 | 0.3638 |
| CVD mortality |  |  |  |

NAFLD, non-alcoholic fatty liver disease; CDAI, Composite dietary antioxidant index.

Table S6. Association of CDAI with prevalence of NAFLD excluding those with a history of alcohol consumption

|  | Crude model | Model 1 | Model 2 |
| --- | --- | --- | --- |
|  | OR (95% CI) | OR (95% CI) | OR (95% CI) |
| CDAI, continuous | 0.97 (0.96, 0.98) | 0.98 (0.97, 0.99) | 0.98 (0.96, 0.99) |
| CDAI, quartile |  |  |  |
| Q1 | Reference (1.0) | Reference (1.0) | Reference (1.0) |
| Q2 | 0.95 (0.87, 1.04) | 0.97 (0.88, 1.06) | 0.96 (0.87, 1.07) |
| Q3 | 0.90 (0.82, 0.99) | 0.98 (0.89, 1.07) | 0.96 (0.86, 1.07) |
| Q4 | 0.77 (0.70, 0.84) | 0.85 (0.78, 0.94) | 0.84 (0.74, 0.97) |
| *P* for trend | <0.0001 | 0.0019 | 0.0254 |

NAFLD, non-alcoholic fatty liver disease; CDAI, Composite dietary antioxidant index.

Table S7. Association of CDAI with prevalence of NAFLD excluding those with cancer

|  | Crude model | Model 1 | Model 2 |
| --- | --- | --- | --- |
|  | OR (95% CI) | OR (95% CI) | OR (95% CI) |
| CDAI, continuous | 0.97 (0.96, 0.98) | 0.98 (0.97, 0.99) | 0.98 (0.96, 0.99) |
| CDAI, quartile |  |  |  |
| Q1 | Reference (1.0) | Reference (1.0) | Reference (1.0) |
| Q2 | 0.95 (0.87, 1.03) | 0.97 (0.89, 1.06) | 0.98 (0.89, 1.08) |
| Q3 | 0.87 (0.80, 0.95) | 0.97 (0.88, 1.05) | 0.99 (0.89, 1.10) |
| Q4 | 0.75 (0.69, 0.82) | 0.86 (0.79, 0.94) | 0.91 (0.80, 1.03) |
| *P* for trend | <0.0001 | 0.0009 | 0.2036 |

NAFLD, non-alcoholic fatty liver disease; CDAI, Composite dietary antioxidant index; CVD, cardiovascular disease.
